# Deep-sea salt as a novel additive for 3D-printed surimi: boosting protein bonding, antioxidant capacity, and digestibility

**DOI:** 10.1016/j.fochx.2025.102942

**Published:** 2025-08-21

**Authors:** Yaqin Hu, Zijing Lu, Zhiheng Hu, Guangyu Liu, Gaoshang Li, Jiayin Huang, Yaoxian Chin, Chunhong Yuan, Dongxue Wang

**Affiliations:** a College of Food Science and Engineering, Yazhou Bay Innovation Institute, Hainan Tropical Ocean University, Marine Food Engineering Technology Research Center of Hainan Province, Collaborative Innovation Center of Marine Food Deep Processing, Hainan Key Laboratory of Herpetological Research, Sanya 572000, China; b United Graduate School of Agricultural Sciences, Ueda 3-8-18, Morioka, Iwate 020-8550, Japan c Faculty of Agriculture, Iwate University, Ueda 3-8-18, Morioka, Iwate 020-8550, Japan; c School of Food Science and Engineering, Ningbo University, Ningbo 315800, Zhejiang, China; d College of Ocean Food and Biological Engineering, Jimei University, Xiamen 361021, China

**Keywords:** Deep-sea salt, Surimi product, 3D printing, Response surface, Microstructure, Chemical bonding

## Abstract

Enhancing both structural integrity and nutritional properties is crucial for developing a functional three-dimensional (3D)-printed surimi formulation. Herein, deep-sea salt was used as a substitute for conventional salt to develop 3D-printed surimi. The physicochemical properties, sensory scores, microstructural examinations, chemical bonding analysis, digestion studies, and antioxidant activity of the 3D-printed surimi were systematically evaluated. The results indicated that the 3D-printed surimi was formulated with the optimal proportions of deep-sea salt (1.5 %), rice starch (2.0 %), and lutein (0.5 %). Compared with the conventional salt, use of deep-sea salt altered the intermolecular interactions within surimi, thereby increasing ionic, hydrogen, and disulfide bonds by 68.3 %, 41.4 %, and 6.2 %, respectively, which increased gel strength by 38.8 %. In addition, deep-sea salt enhanced the antioxidant capacity and in vitro digestibility of surimi, increasing the latter from 64.2 % to 70.7 %. These results indicate that deep-sea salt enhances the nutritional functionality of 3D-printed surimi, making it a valuable additive for the development of functional seafood products.

## Highlights

• We prepared and optimized a 3D-printed surimi formula.
• Deep-sea salt boosted ionic/hydrogen bonds, enhancing gel strength.
• Deep-sea salt formed a dense, uniform protein network, enhancing structural support.
• Deep-sea salt boosted printing performance and nutritional functionality.

## 1 Introduction

3D printing being a cutting-edge technology, is widely applied in food, aerospace, biology and medicine, and construction manufacturing domains (Wu et al., 2015). Within the food industry, this technology has mainly been used for the production of items, such as chocolates, surimi, candies, cheeses, and various pastes (Ishigaki et al., 2018), offering various advantages, such as mold-free rapid prototyping and personalized designs (Dick et al., 2020). Therefore, 3D food printing aligns with the development trends of future food production systems, demonstrating considerable potential for customization, efficiency, and innovation.

The silver carp (*Hypophthalmichthys molitrix*), a widely distributed freshwater species in China, has become a valuable resource for surimi-based products owing to its high protein content, affordability, and excellent gel-forming properties (Wang et al., 2024). These attributes make it an ideal material for value-added food innovation, which addresses the growing demand for cost-effective protein sources and the challenge of global food security. However, conventional processing techniques often struggle to deliver products with tailored textures, structures, and nutritional profiles that could satisfy diverse consumer preferences. Herein, the advent of 3D food printing technology offers a transformative approach to surimi processing. For example, Li et al. (2023a) infused nano-starch and lutein into surimi, to enhance its nutritional value and facilitated the development of nutrient-functional 3D-printed foods. Similarly, Liu et al. (2021) reported that the addition of sweet potato starch could enhance the gel strength of golden pomfret surimi and effectively improve its gel properties and quality. Addition of starch—an auxiliary material to final surimi products—is essential as its addition affects the overall quality of surimi processing.

To meet the specific rheological and curing requirements for 3D printing materials, additive innovations are crucial. Deep sea water (DSW) is rich in trace minerals, such as magnesium, calcium, and potassium, as reported by Lee (2015). A balanced intake of there minerals is essential for maintaining human health. The main sources of daily mineral intake include food and drinking water (Shiraishi et al., 2017). Therefore, deep-sea salt extracted from DSW has a unique mineral matrix in comparison with conventional salt. These attributes not only meet consumer expectations for nutritional enhancement but can also enhance the structural integrity and nutritional profile of surimi. Collectively, these advancements present a promising framework for leveraging underutilized aquatic resources, such as silver carp, to develop innovative, nutrient-dense, and environmentally friendly food solutions.

Herein, deep-sea salt, rice starch, and lutein were used as major additives for preparing 3D-printed surimi materials. The quality of the 3D-printed surimi was evaluated by examining its physicochemical properties and sensory attributes. Optimal preparation parameters were established through single-factor experiments and response surface methodology (RSM). The effects on surimi structure were systematically analyzed via scanning electron microscopy (SEM), chemical bonding, and texture analysis. In addition, alterations in its functional properties were assessed via antioxidant activity and in vitro digestion tests. Herein, we aimed to optimize a 3D-printed surimi material infused with deep-sea salt to highlight its potential application within the surimi industry for the development of new value-added surimi products.

## 2 Materials and methods

### 2.1 Materials

Rice starch was obtained from Shanghai Yuanye Biotechnology Co., Ltd. (Shanghai, China). Deep ocean water was sourced from YES Deep Water Company, Hualien, Taiwan (Taiwan, China). Lutein was procured from Shandong Ruian Biotechnology Co., Ltd. (Shandong, China). Surimi was supplied by Honghu Jingli Aquatic Food Co., Ltd. (Hubei, China); it was transported to the laboratory on ice and stored at −80 °C upon arrival. Kits for ABTS and DPPH free radical scavenging assays were purchased from Beijing Solarbio Technology Co., Ltd. (Beijing, China), while the total antioxidant capacity assay kit was obtained from Shanghai Xinyu Biotechnology Co., Ltd. (Shanghai, China). Pepsin and trypsin were acquired from Beijing Sinopharm Chemical Reagent Co., Ltd. (Beijing, China). All other chemical reagents used in this study were of analytical grade.

### 2.2 Single-factor experiment

The amounts of rice starch, deep-sea salt, and lutein were taken in different ratios to prepare 3D-printed surimi materials. A single-factor experimental design was used to explore their individual effects on 3D printing performance, with the physical, chemical, and sensory properties of the printed surimi products used as evaluation criteria. The amount of material to be added to the surimi was determined according to the method described by Li et al. (2023b) and Cao et al. (2022), with slight modifications. All experiments were conducted in three independent replicates (*n* = 3), maintaining consistent printing parameters throughout.

#### 2.2.1 Effect of rice starch

Surimi was processed in a grinder, and rice starch was added at concentrations of 0 %, 0.5 %, 1.0 %, 2.0 %, 4.0 %, and 8.0 %. After addition at each concentration, the starch was gently mixed with surimi. Deep-sea salt (1.0 %) and lutein (0.5 %) were consistently added across all groups. The prepared mixtures were loaded into barrels for 3D printing.

#### 2.2.2 Effect of deep-sea salt

Surimi was processed in a grinder, and deep-sea salt was added at concentrations of 0 %, 0.5 %, 1.0 %, 1.5 %, 2.0 %, and 2.5 %. Each addition was followed by gentle mixing. Across all groups, the rice starch content was controlled at 2.0 % and lutein was fixed at 0.5 %. The prepared mixtures were loaded into barrels for 3D printing. Each condition was tested in triplicate.

#### 2.2.3 Effect of lutein

Minced surimi was infused with lutein at concentrations of 0 %, 0.25 %, 0.50 %, 0.75 %, 1.00 %, and 1.25 %. The rice starch content was maintained at 2 %, and deep-sea salt concentration was fixed at 0.5 %. The resulting mixtures were loaded into barrels for 3D printing. Each condition was tested in triplicate under identical experimental settings.

### 2.3 Response surface experiment

The RSM was implemented using the Box–Behnken design. Experimental design, model analysis, and statistical evaluations were performed using the Design-Expert 13.0 software (Stat-Ease, Inc., Minneapolis, USA). Table 1 presents the experimental factors and levels for response surface optimization. The independent variables included the added concentrations of deep-sea salt, rice starch, and lutein, while the response variable was the sensory score.

**Table 1:** Experimental factors for response surface optimization.

Levels	Factors		
	A Rice starch (%)	B Deep-sea salt (%)	C Lutein (%)
−1	1.5	1.0	0.2
0	2.0	1.5	0.5
1	2.5	2.0	0.8

### 2.4 Sensory evaluation

Sensory evaluation was performed according to the method described by Li, Chen, et al. (2019), with minor modifications. Evaluations were conducted under consistent conditions by a sensory assessment panel comprising 12 trained assessors. All participants were informed of the detailed content of this study, and they consented to participate and granted permission for the use of data generated during the experiment. The evaluation indices, as presented in Table 2, included texture, elasticity, odor, and appearance.

**Table 2:** Sensory evaluation indices for surimi.

Evaluation index	Sensory score			
	9–10	6–8	3–5	0–2
Texture	The surface is smooth, the tissue structure is compact and uniform with a well-defined texture	The surface is smooth, the tissue structure is dense with a well-defined texture	Small pores are observed on the surface, the tissue structure is relatively loose	The surface structure is not compact and has a loose and soft texture
Elasticity	Very elastic, has superior compression recovery	Elastic, has good compression recovery	Has average elasticity and compression recovery	Soft and inelastic, has slow compression recovery
Odor	Balanced fish flavor with no off-odor	Insufficient fish aroma with a slight fishy odor	No fish aroma, too fishy	No fish aroma, off-odor
Appearance	Uniform color with a glossy surface	The color is slightly dull with an average surface luster	Dull color, with no obvious surface luster	Dark color, with a lackluster surface

### 2.5 Determination of texture properties

The texture properties of the 3D-printed surimi products were evaluated using the method described by Li et al. (2023a). Texture profile analysis (TPA) was performed using a texture analyzer in the TPA mode. A P-50 probe was used with a vertical movement distance of 30 mm. The pretest, test, and post-test speeds were set at 1.0 mm/s, with a deformation rate of 50 %, a trigger force of 5 gf, and a 5-s interval between the tests.

### 2.6 Determination of gel strength

The gel strength of the 3D-printed surimi products was determined using the method described by Li et al. (2023a). The texture analyzer was operated in a single test mode using a P-0.25 probe with a vertical movement distance of 30 mm. The pretest, test, and post-test speeds were all set at 1.0 mm/s, with a deformation rate of 50 %, a trigger force of 5 gf, and a 5-s interval between the tests. Gel strength (N·mm) was calculated as the product of the maximum force (N) and the corresponding compression distance (mm).

### 2.7 Measurement of rheological properties

The rheological properties of the 3D-printed surimi products were evaluated using a rheometer to perform frequency and strain sweeps. Frequency sweeps were performed over a range of 0.1–100 Hz at a constant strain of 1 %, generating viscosity curves. Subsequently, strain sweeps were performed with a shear stress range of 0.1–1000 Pa to determine the modulus–shear stress relationship.

### 2.8 Test of antioxidant property

ABTS radical scavenging activity was determined using an ABTS radical cation decolorization assay kit (Beijing Solarbio Technology Co., Ltd., Beijing, China). DPPH radical scavenging activity was evaluated using a DPPH radical scavenging assay kit (Beijing Solarbio Technology Co., Ltd., Beijing, China). The ferric reducing antioxidant power (FRAP) was assessed using a FRAP assay kit (Shanghai Xinyu Biotechnology Co., Ltd., Shanghai, China).

### 2.9 Observation of microstructure

The sample microstructure was characterized via SEM according to the protocol described by Kubota et al. (2003), with slight modifications. Freeze-dried samples were mounted on double-sided conductive tape and spray-coated with gold particles. Micrographs were obtained using a field emission scanning electron microscope (JSM-7610F PLUS; JEOL Ltd., Tokyo, Japan) operated at an accelerating voltage of 10 kV and a working distance of 8.0 mm.

### 2.10 Determination of chemical bonds

The chemical bonds of the samples were analyzed according to the method described by Li, Wu, et al. (2019). Five distinct solutions were prepared by combining 1 g of the sample with 5 mL of each solutions as follows: A (0.05-mol/L NaCl), B (0.6-mol/L NaCl), C (0.6-mol/L NaCl and 1.5-mol/L urea), D (0.6-mol/L NaCl and 8-mol/L urea), and E (0.6-mol/L NaCl, 8-mol/L urea, and 0.05-mol/L β-mercaptoethanol). Following incubation at 4 °C for 1 h and centrifugation at 12,500 ×*g* for 15 min, the protein content in the supernatant was determined using the Folin-phenol method. The relative contributions of ionic, hydrogen, and disulfide bonds as well as hydrophobic interactions were estimated by comparing the protein content of surimi extracted using solution B versus solution A, C versus B, D versus C, and E versus D.

### 2.11 Assay of in vitro digestion

The in vitro digestibility of the samples was evaluated following the method described by You et al. (2022) with slight modifications. The initial weight of the matured sample (M_1_) was recorded, and the sample was subsequently homogenized with water. The pH of the resulting suspension was adjusted to 2.0, and pepsin was added to initiate the digestion process. The mixture was incubated at 37 °C with gentle shaking at 100 rpm for 1 h. Subsequently, the pH was adjusted to 7.0 and trypsin was added to simulate intestinal digestion under the same temperature conditions for 2 h, with the shaking speed maintained at 100 rpm. The reaction was terminated by heating the samples in a water bath at 90 °C for 15 min. Following with the enzyme inactivation, the samples were centrifuged at 5000 rpm for 5 min. Furthermore, the supernatant was decanted and the weight of the resulting precipitate (M_2_) was recorded. In vitro digestibility was calculated as follows: [(M_1_ − M_2_) / M_1_] × 100 %.

### 2.12 Statistical analysis

All measurements were performed in triplicate. Data were expressed as mean ± standard deviation and analyzed using IBM SPSS Statistics 26 (IBM Corp., Armonk, New York, USA). Figures and tables were generated using Origin Pro 2021 (Origin Lab, Northampton, Massachusetts, USA). Statistical significance was determined via one-way analysis of variance (ANOVA) and response surface analysis at a significance level of *P* < 0.05.

## 3 Results and discussion

### 3.1 Influence of single factors on 3D-printed surimi

**Fig. 1:**
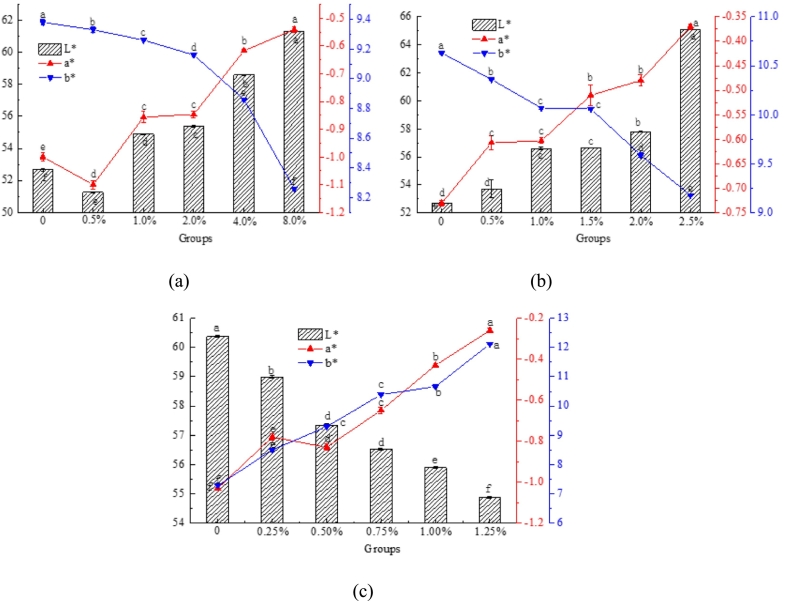
Color differences in 3D-printed surimi in the following scenarios: (a) rice starch with different contents, (b) deep-sea salt with different contents, and (c) lutein surimi with different contents. Different letters indicate significant differences (*P* < 0.05).

The results presented in Fig. 1a and b indicate that the increase in rice starch and deep-sea salt concentrations enhanced the brightness and diminished the yellowness of the surimi materials. Conversely, the addition of lutein reduced the L* value and increased the a* and b* values in the surimi samples (Fig. 1c), indicating a more intense red coloration and enhanced color vibrancy, thereby improving the visual appeal (Li et al., 2023a).

**Fig. 2:**
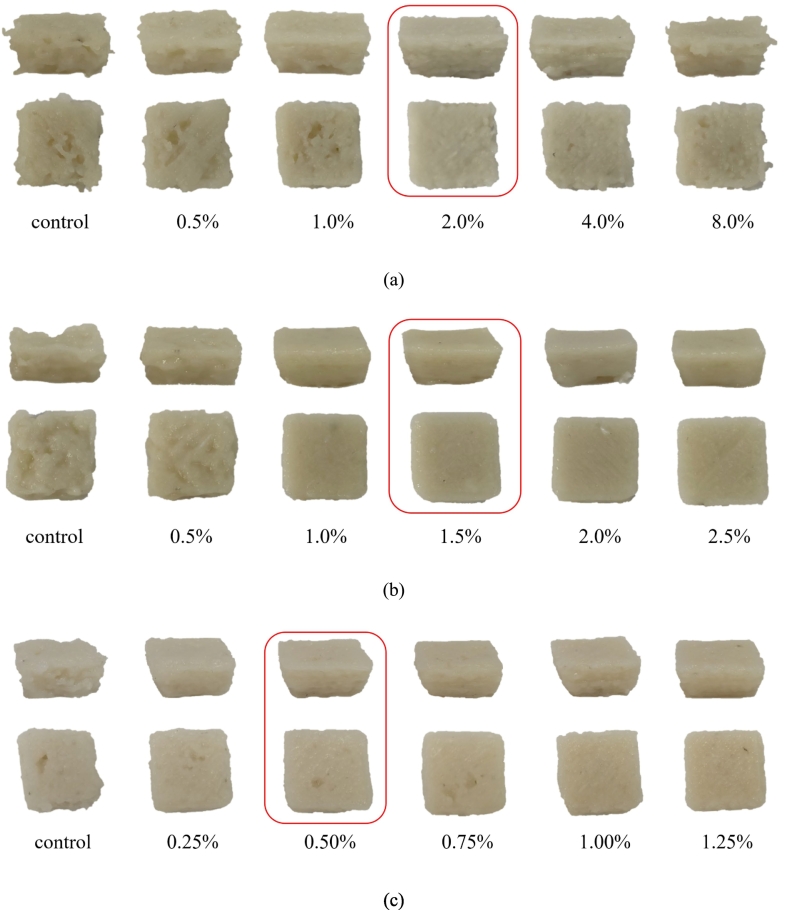
Pictures of 3D-printed surimi under the following conditions: (a) rice starch with different contents, (b) deep-sea salt with different contents, and (c) lutein infused surimi with different contents.

**Fig. 3:**
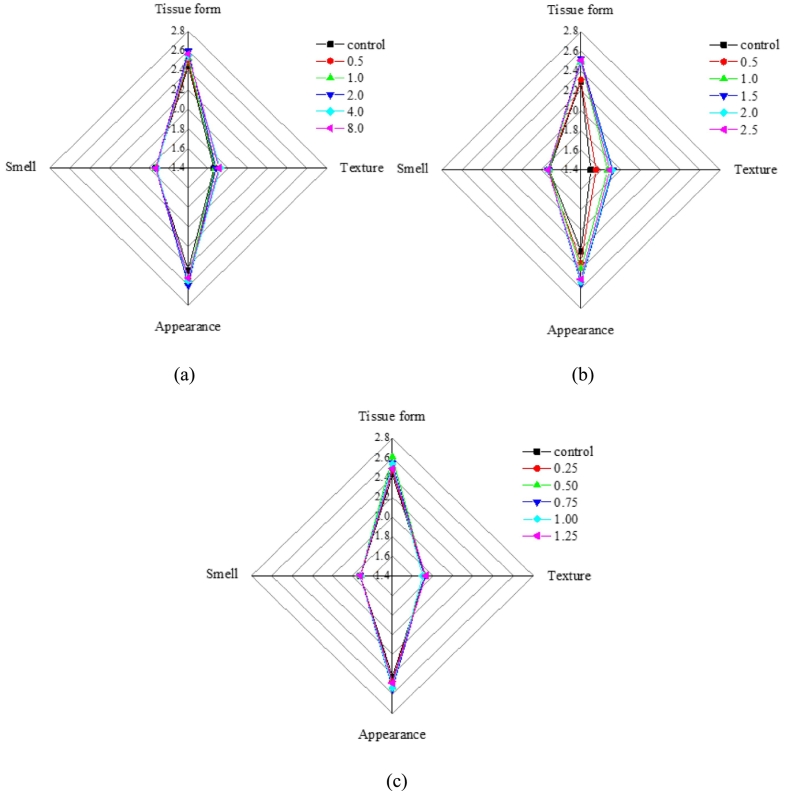
Results of sensory evaluation under the following conditions: (a) rice starch with different contents, (b) deep-sea salt with different contents, and (c) lutein infused surimi with different contents.

The optimal sensory scores for the 3D-printed surimi were achieved with the following addition concentrations: 2.0 % rice starch (Figs. 2a and 3a), 1.5 % deep-sea salt (Figs. 2b and 3b), and 0.5 % lutein (Figs. 2c and 3c). Under these conditions, the surimi demonstrated a smoother and more intact surface and structure, resulting in enhanced printing fidelity.

### 3.2 Texture and gel-strength characteristics of surimi

**Fig. 4:**
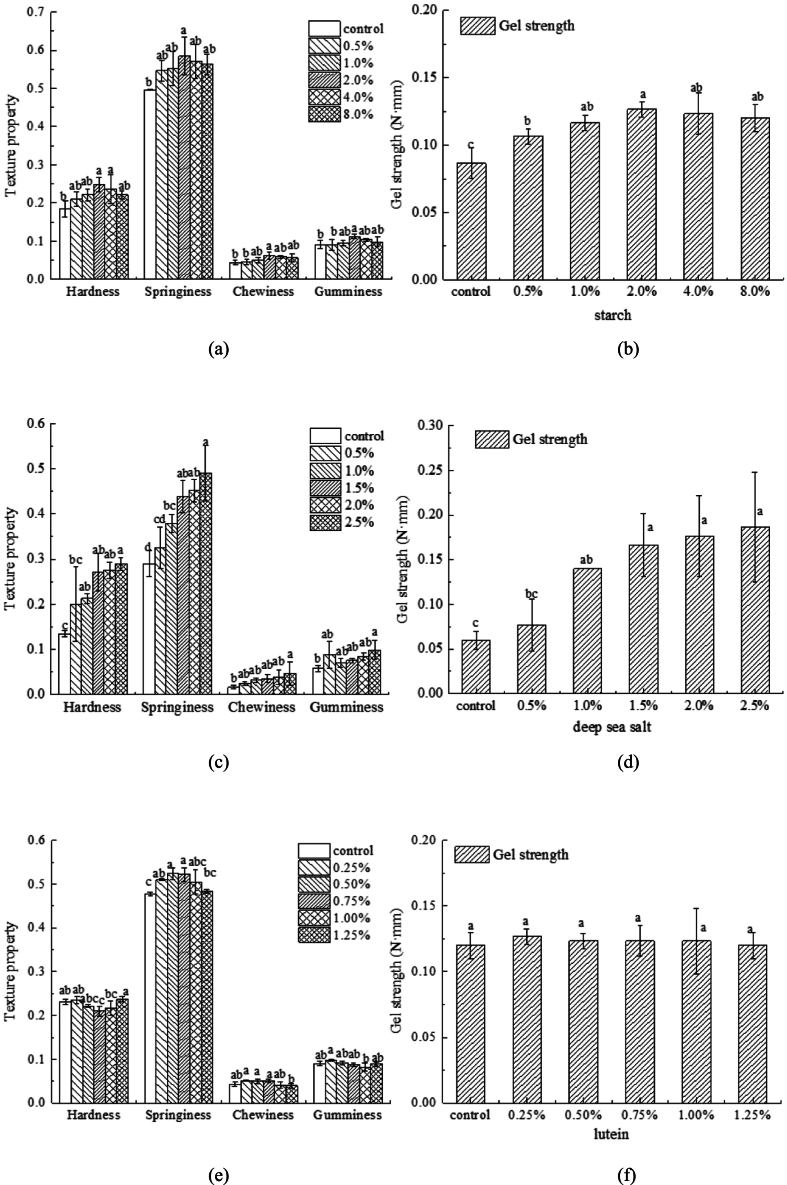
Textural and gel characterization of 3D-printed surimi. (a) The influence of the different concentrations of rice starch on texture and (b) on gel strength; (c) The influence of different concentrations of deep-sea salt on texture and (d) on gel strength; (e) The influence of different concentrations of lutein on texture and (f) on gel strength. Different letters indicate significant differences (*P* < 0.05).

The results presented in Fig. 4b indicate that the gel strength of surimi initially increases and then decreases with increasing rice starch content, reaching its peak at an addition concentration of 2 %. Gel strength and other textural properties are important factors for the successful 3D printing of surimi (Mi et al., 2021). At this optimal concentration, the printed and extruded materials exhibited greater mechanical strength and structural support compared with the other groups, demonstrating resistance to collapse and deformation post-printing. This enhancement is attributed to the rice starch embedding within the gel matrix, which reinforces cross-linking and promotes protein aggregation, consistent with the findings of Luo et al. (2020) and Yanmo Pan et al. (2021). In essence, rice starch reinforces intermolecular interactions via inducing gelation within the surimi matrix. This increased molecular binding subsequently enhances the textural properties of surimi, as reported by Liu et al. (2014).

As the salt concentration increased, the texture of surimi enhanced, concurrently increasing the strength and overall quality of the gel (Fig. 4). Previous studies have demonstrated that the addition of salt influences intra- and intermolecular interactions as well as covalent cross-linking of surimi proteins (Liang et al., 2020). This effect could be attributed to the dissolution of salt-soluble proteins upon salt addition, which enhances the hydrogen bonding between surimi molecules, thereby facilitating the formation of a robust gel network and subsequently strengthening the cross-linking within the protein structure. These modifications can facilitate 3D printing and enhance printability (Feng et al., 2022).

### 3.3 Rheological properties of surimi

The shear stress at the intersection of the storage and loss moduli is a key indicator of the extrusion pressure required for 3D printing (Chen, Mu, et al., 2019; Dong et al., 2023). Proper rheological properties are essential for ensuring effective curing and molding of surimi-based materials (Chen et al., 2022).

**Fig. 5:**
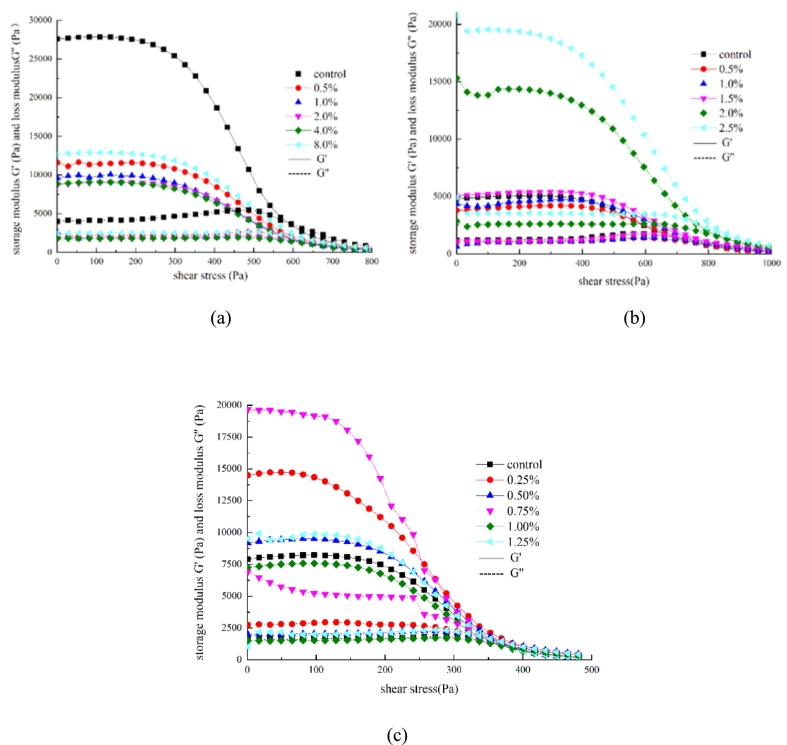
Shear stress modulus of 3D-printed surimi. (a) Rice starch with different contents, (b) deep-sea salt with different contents, and (c) lutein infused surimi with different contents.

Results indicated that shear stress increased with the addition of deep-sea salt and rice starch, indicating an increase in the amount of insoluble particles that could contribute to localized blockages and higher extrusion forces (Fig. 5a and b). While excessive shear stress may cause material accumulation owing to over-extrusion, it enhances post-printing stability (Carvajal-Mena et al., 2022; Zhu et al., 2019). These findings confirm that both the additives enhance protein cross-linking and gel density. Nevertheless, lutein could improve rheological behavior and facilitate smoother extrusion (Fig. 5c). In conclusion, the incorporation of rice starch, deep-sea salt, and lutein modulates the rheological properties, extrusion behavior, and moldability of surimi, supporting enhanced 3D printing performance.

### 3.4 Response surface optimization experiment

Based on the results of single-factor experiments, RSM was used to optimize the formulation, with the sensory score (Y) of surimi used as the response variable and the added concentrations of rice starch (A), deep-sea salt (B), and lutein (C) as the independent variables. Table S4 presents in detail the factor levels used for RSM optimization. The experimental design and corresponding results of RSM optimization are shown in Table S5. Regression analysis of the data revealed the following quadratic polynomial equation: Y = 8.68 + 0.0094 A + 0.0106B + 0.0100C − 0.0125AB + 0.0012 AC − 0.0237 BCE − 0.2013A^2^ − 0.4363B^2^ − 0.3675C^2^. The data were subjected to ANOVA, as shown in Table S5, and the results are summarized in Table S6. The model demonstrated high significance (*P* < 0.0001) value, with a high coefficient of determination (R^2^) of 0.9995, indicating a good fit. The lack of fit test was nonsignificant (*P* > 0.05), confirming a strong agreement between the model and the observed response surface (Akram & Garud, 2020). The ANOVA results revealed that all three factors (A, B, and C) and the interaction terms (AB and BC) exerted considerable effects on the sensory scores of surimi (*P* < 0.05). The relative influence of each factor on the sensory scores can be ordered as follows: deep-sea salt > lutein > rice starch.

**Fig. 6:**
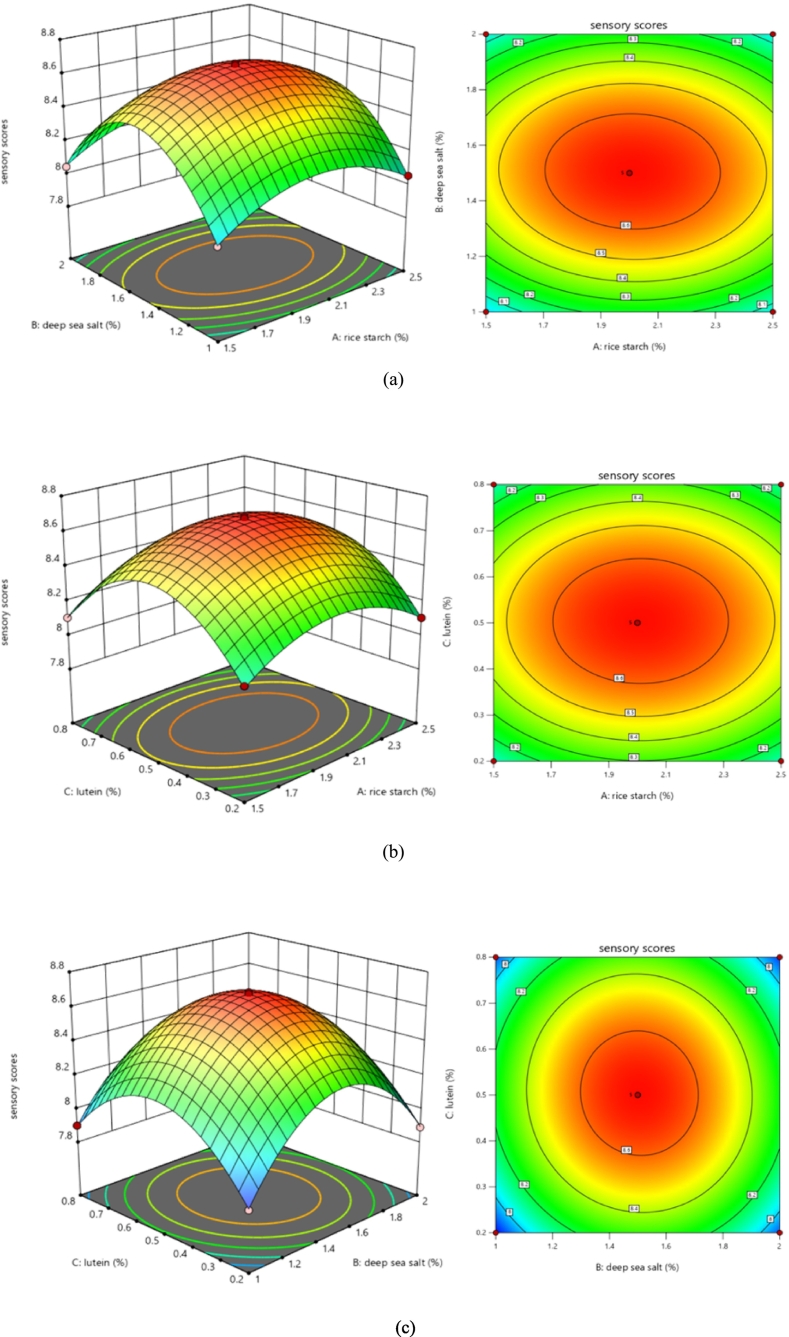
Impact of deep-sea salt and lutein addition on the sensory scores of surimi. (a) Impact of rice starch and deep-sea salt addition of surimi. (b) Impact of rice starch and lutein addition of surimi. (c) Impact of deep-sea salt and lutein addition of surimi.

According to the response surface and contour plot analyses, steeper response surface slopes and more elliptical contour lines indicate a stronger interaction between factors (Hu et al., 2022). As shown in Fig. 6a, the interaction between rice starch and deep-sea salt was considerable. Contrarily, the interaction between rice starch and lutein was insignificant (Fig. 6b). Meanwhile, the interaction between deep-sea salt and lutein was significant (Fig. 6c).

**Fig. 7:**
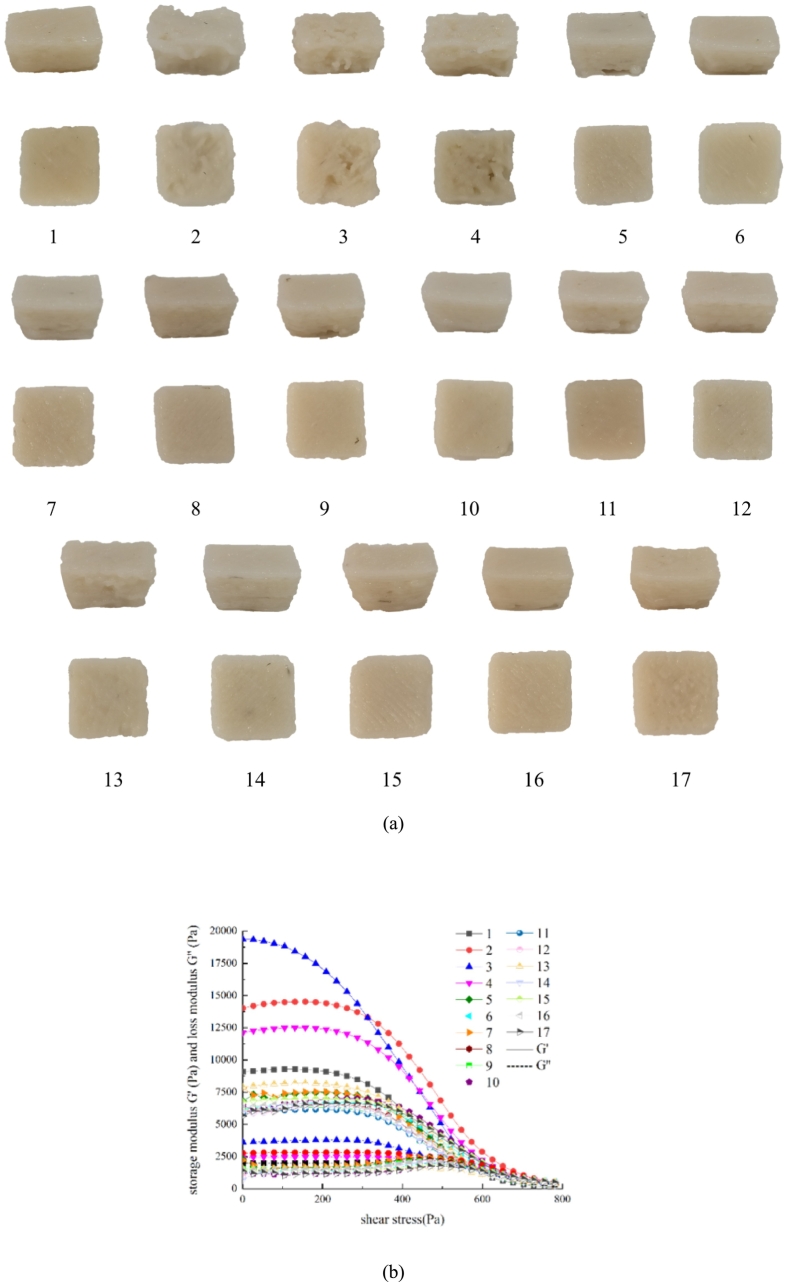
Pictures and shear stress modulus of surimi by response surface optimization experiments. (a) Pictures of 3D-printed surimi. (b) Shear stress modulus of 3D-printed surimi.

As presented in Fig. 7a, groups 8, 9, and 14 demonstrated desirable color characteristics, along with optimal 3D printing molding and structural integrity (Fig. 7b). The results of the response surface experiments and ANOVA indicated that the optimal added concentrations for 3D-printed surimi were determined using the Design-Expert software. However, for practical applications, the component levels were rounded to 2.0 % rice starch, 1.5 % deep-sea salt, and 0.5 % lutein. At these adjusted concentrations, the achieved sensory score was 8.675.

### 3.5 Changes in the chemical bond properties and microstructure of surimi

**Fig. 8:**
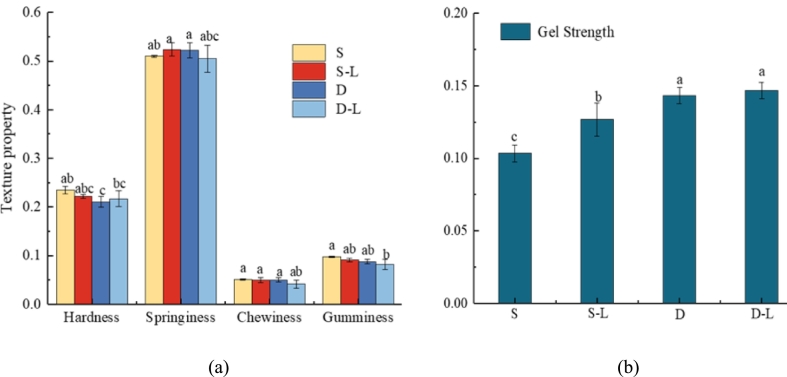
Textural properties and gel strength of different 3D-printed surimi are as follows: S, salt surimi; S–L, salt lutein surimi; D, deep-sea salt surimi; and D–L, deep-sea salt lutein surimi. (a) Textural properties of the different types of 3D-printed surimi and (b) gel strength of the different types of 3D-printed surimi. Different letters indicate significant differences (*P* < 0.05).

To further explore the effects of deep-sea salt, a control group using commercial salt was included for comparison with the 3D-printed surimi produced under optimized conditions. Deep-sea salt markedly increased gel strength by 38.8 % compared with the control (Fig. 8a and b). This enhancement was attributed to the ability of deep-sea salt to fill voids within the protein gel network, thereby providing structural support to the mesh. Consequently, this enhanced the mechanical strength and structural integrity of the 3D-printed surimi (Xu et al., 2023). Subsequently, the density of the surimi protein gel network increased, which led to improved gel strength and textural properties in the 3D-printed surimi (Bao Yirui et al., 2021), as well as enhanced structural support and bonding capacity (Chen, Xie, et al., 2019).

**Fig. 9:**
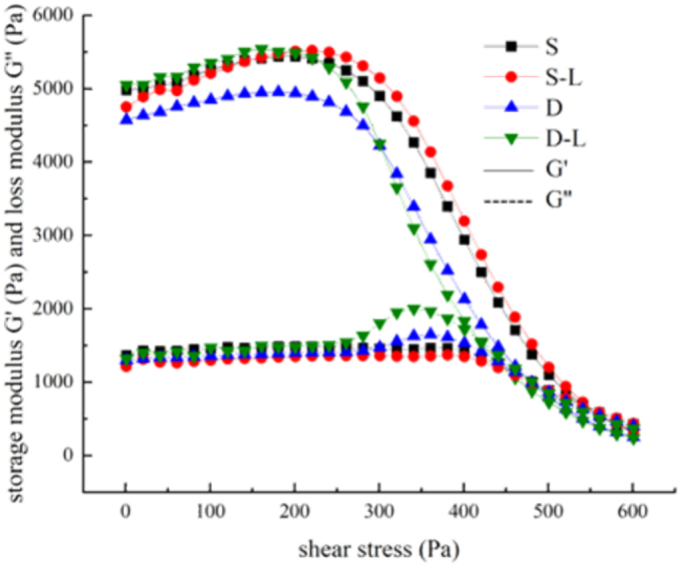
Comparison of shear stress modulus among the different types of 3D-printed surimi is as follows: S, salt surimi; S–L, salt lutein surimi; D, deep-sea salt surimi; and D–L, deep-sea salt lutein surimi.

**Fig. 10:**
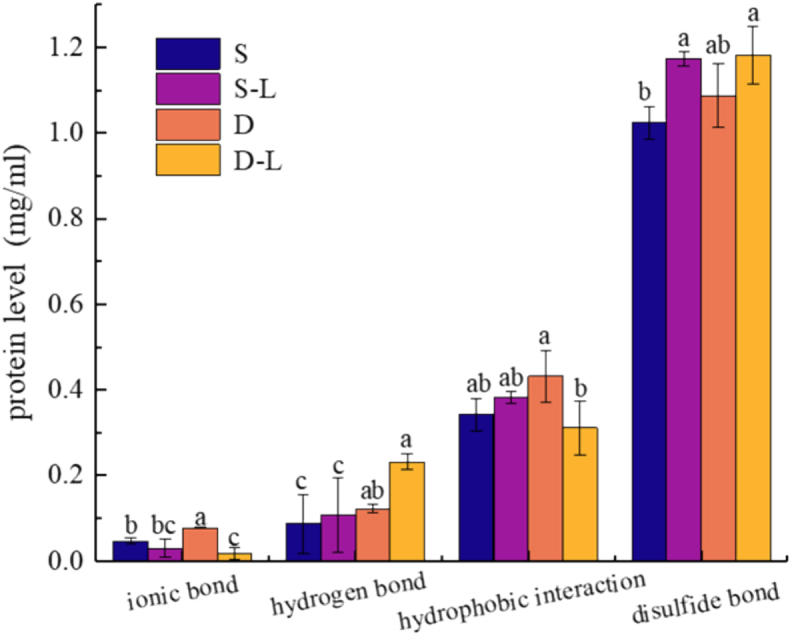
Analysis of chemical bonds in the different types of 3D-printed surimi is as follows: S, salt surimi; S–L, salt lutein surimi; D, deep-sea salt surimi; and D–L, deep-sea salt lutein surimi. Different letters indicate significant differences (*P* < 0.05).

Deep-sea salt–treated samples showed enhanced flowability and toughness (Fig. 9). This enabled them to better resist fracture during tensile stress and reduced material shear stress. It was hypothesized that the enhanced toughness was related to changes in chemical bonding. Subsequent analysis of chemical bonds in the printed materials (Fig. 10) revealed that deep-sea salt increased the number of ionic, hydrogen, and disulfide bonds within the surimi. It is speculated that the surimi gelation process is time-dependent (Zheng et al., 2022). The increase in hydrogen bonds likely stabilizes the secondary structure of the proteins, thereby preventing structural breakdown and thus improving gel strength while enhancing water binding and retention. The increase in the number of disulfide bonds facilitates intramolecular polymerization, leading to a denser protein gel structure and enhancing mechanical strength (Li et al., 2023b). The increased ionic bonds promoted the electrostatic interaction between molecules, resulting in a more compact material structure, enhanced resistance to deformation and fracture as well as improved elastic modulus and self-supporting ability (Zhao et al., 2021).

**Fig. 11:**
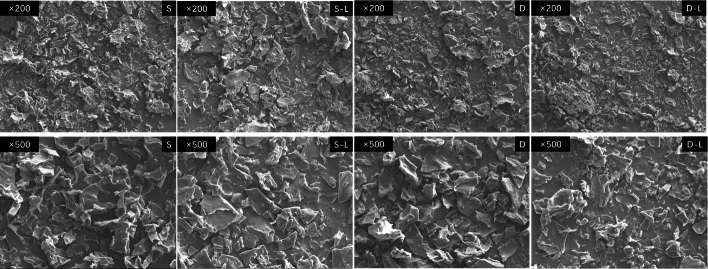
Microstructure of 3D-printed surimi is as follows: S, salt surimi; S–L, salt lutein surimi; D, deep-sea salt surimi; and D–L, deep-sea salt lutein surimi.

SEM analysis of the surimi microstructure (Fig. 11) revealed that samples infused with deep-sea salt exhibited smaller particle sizes and more uniform particle distributions. This difference is likely due to the promotion of homogeneity and dense protein network by deep-sea salt.

### 3.6 Correlation analysis among the key quality indicators of 3D-printed surimi

**Fig. 12:**
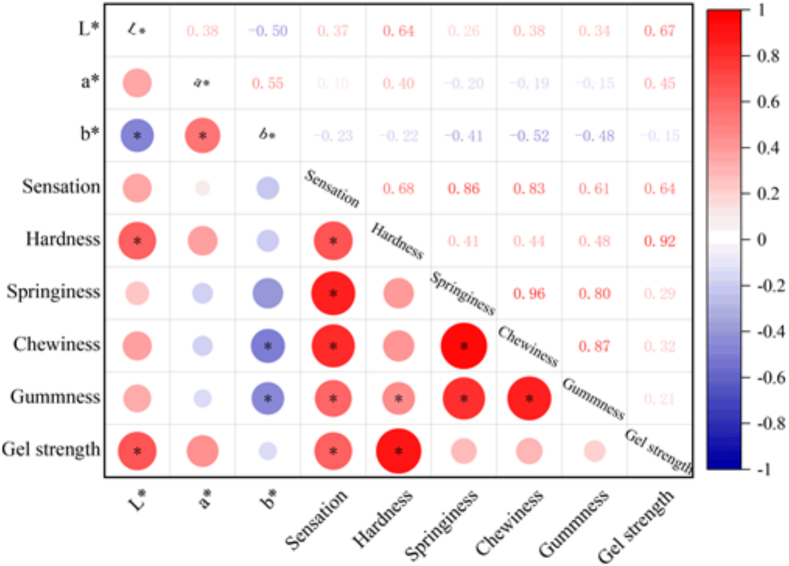
Correlation analysis among the key quality indicators of 3D-printed surimi. * represented significant correlation (P < 0.05).

Gel strength showed strong correlation with hardness (*r* = 0.92), sensory score (*r* = 0.64), and L* brightness (*r* = 0.67) (Fig. 12), confirming its potential in achieving printability and consumer acceptance. In the context of 3D printing, gel strength modulates performance through two main mechanisms: it enhances hardness and self-supporting capacity to ensure dimensional accuracy, and excessive elevation increases extrusion resistance (Zhang et al., 2021). Consequently, optimal gel strength must be maintained within a defined range to balance printability and structural integrity.

Regarding textural properties, elasticity, chewiness, and gumminess form a highly integrated network (*r* ≥ 0.80). Notably, the correlation between elasticity and chewiness (*r* = 0.96) as well as between chewiness and gumminess (*r* = 0.87) indicates a self-reinforcing relationship that collectively influences sensory perception. Conversely, color parameters exert antagonistic effects: L* positively correlates with gel strength and hardness but inversely affects b* (*r* = −0.50). Similarly, a* positively correlates with gel strength (*r* = 0.45) but impairs elasticity (*r* = −0.20). However, lutein may compensate for yellowness reduction at high brightness levels, enabling coordinated optimization of texture, print quality, and visual properties.

### 3.7 In vitro digestion and antioxidant of D-L surimi

**Fig. 13:**
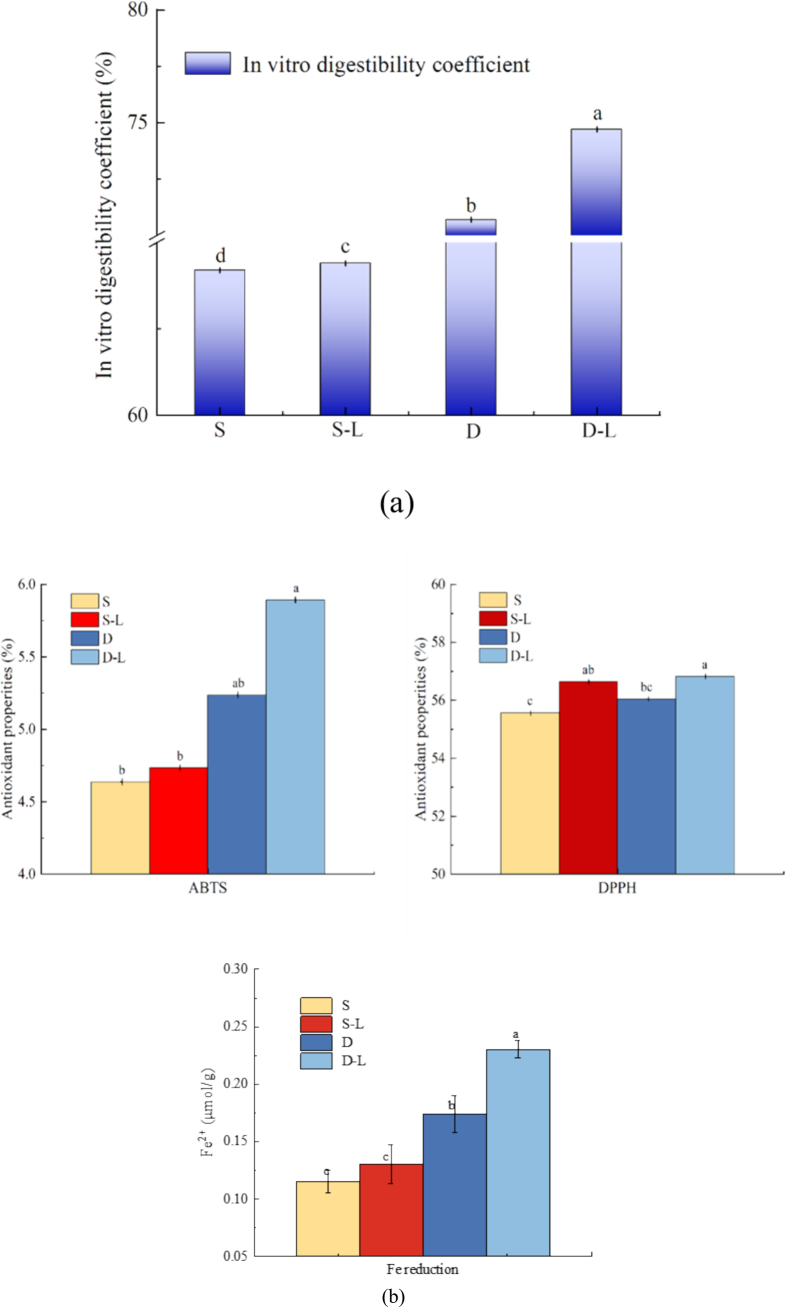
In vitro digestibility coefficients and antioxidative assays of the following 3D-printed surimi: S, salt surimi; S–L, salt lutein surimi; D, deep-sea salt surimi; and D–L, deep-sea salt lutein surimi. (a) In vitro digestibility coefficients of 3D-printed surimi and (b) antioxidative assays of 3D-printed surimi. Different letters indicate significant differences (*P* < 0.05).

Aside from its impact on texture and gel strength, in vitro digestibility assays revealed that surimi supplemented with deep-sea salt demonstrated considerably higher digestibility compared with the control groups (Fig. 13a). This is attributable to the distinct mineral composition and structural properties of deep-sea salt, which facilitate more effective protein cross-linking and gel network formation. Unlike conventional salt, deep-sea salt contains trace minerals, such as magnesium, calcium, and other elements, which may enhance protein stability and interaction, resulting in superior quality gel matrix development and reinforcing the structural network. Studies have demonstrated that the dense, porous, three-dimensional gel network formed via protein cross-linking increases the porosity of the protein structure, thereby facilitating the binding of digestive enzymes to proteins and promoting their hydrolysis (Xiao et al., 2020; Yuris et al., 2019). In addition, deep-sea salt may inherently enhance enzyme activity and promote hydrolysis during digestion, further contributing to the increased digestibility. This superior effect differentiates deep-sea salt from conventional salts and highlights its functional benefits for nutrient absorption.

Lutein-enriched deep-sea salt surimi demonstrated considerably higher ABTS, DPPH, and FRAP values than its conventional salt counterpart (Fig. 13b). These results indicate that the antioxidant capacity of lutein remained intact and effective, even in the presence of deep-sea salt. This could be attributed to the protein matrix formed by deep-sea salt, which appears to encapsulate lutein and facilitate its gradual release (Zhao et al., 2022). Meanwhile, ions from deep-sea salt can form weak coordinate bonds with the hydroxyl groups of lutein, creating stable molecular conformations that help preserve its free radical scavenging ability. Based on a comprehensive analysis of microstructure, chemical bonds, digestion, and antioxidant activity, it is evident that deep-sea salt not only improved the printing characteristics but also markedly augmented the nutritional functionality and overall quality of the 3D-printed surimi, particularly when compared with the products produced using conventional salt.

Overall, deep-sea salt enhances not only the printability of surimi but also its nutritional and bioactive qualities. Its mineral-rich composition fosters a denser gel network, increases digestibility, and preserves bioactive compounds, such as lutein, making it a superior supplement to conventional salt for 3D-printed seafood products.

## 4 Conclusion

This study successfully infused deep-sea salt into 3D-printed surimi, demonstrating dual synergistic mechanisms that enhance structural integrity and nutritional properties. Structurally, deep-sea salt reinforces ionic and hydrogen bonds, forming a dense microstructure that enhances deformation resistance. Nutritionally, trace minerals in the salt synergize with lutein, boosting antioxidant activity and in vitro digestibility. The integration of deep-sea salt into 3D-printed surimi presents a novel strategy for improving food quality, with notable industrial application potential. It enhances printability, structural stability, and nutritional value, making it a promising additive for functional seafood products. In particular, this innovation is impactful for the seafood sector, where deep-sea salt is used as a natural additive to enhance the texture and shelf stability of surimi-based products (e.g., artificial crab, and fish cakes), thereby addressing challenges in mass production and 3D printing scalability. Beyond its direct contributions to the structural and nutritional enhancement of 3D-printed surimi, deep-sea salt's natural origin and high compatibility with the concept of sustainable development, meeting modern consumers' demand for sustainable and environmentally friendly products. Moreover, the observed mineral–antioxidant synergy positions this technology strongly within the functional food market, allowing for the development of nutrient-fortified products, such as elderly-friendly textured foods for health-conscious consumers. Future researchers should prioritize elucidating the detailed mechanisms via which key minerals regulate protein conformation, potentially through molecular dynamic simulations and quantitative structure–activity relationship models. In conclusion, herein, we effectively demonstrated the substantial potential of deep-sea salt to enhance the structural and nutritional aspects of 3D-printed surimi, thereby opening new avenues for innovative food product development.

## CRediT authorship contribution statement

**Yaqin Hu:** Writing – review & editing, Validation, Supervision, Resources, Project administration, Funding acquisition, Conceptualization. **Zijing Lu:** Writing – original draft, Visualization, Software, Methodology, Investigation, Formal analysis, Data curation, Conceptualization. **Zhiheng Hu:** Data curation. **Guangyu Liu:** Methodology. **Gaoshang Li:** Software, Investigation, Formal analysis. **Jiayin Huang:** Investigation. **Yaoxian Chin:** Writing – review & editing. **Chunhong Yuan:** Funding acquisition. **Dongxue Wang:** Validation, Supervision, Resources, Project administration.

## Declaration of competing interest

The authors declare that they have no known competing financial interests or personal relationships that could have appeared to influence the work reported in this paper.

## Data Availability

No data was used for the research described in the article.

## Appendix A Supplementary data

Supplementary material Image 1
