# Supplementary material for: Deep-sea salt as a novel additive for 3D-printed surimi: boosting protein bonding, antioxidant capacity, and digestibility
Source: Food Chem X. 2025 Aug 21;30:102942. doi: 10.1016/j.fochx.2025.102942 (PMC12409813; doi:10.1016/j.fochx.2025.102942)
Supplement: Supplementary file 1 — Supplementary material [file mmc1.pdf]

Deep-sea salt as a novel additive for 3D-printed surimi: boosting protein bonding, antioxidant capacity, and digestibility

Yaqin Hu[a], Zijing Lu[a], Zhiheng Hu[b,a], Guangyu Liu[a], Gaoshang Li[c], Jiayin Huang[d], Yaoxian Chin[a], Chunhong Yuan[b], Dongxue Wang[a*]

[a] *College of Food Science and Engineering, Yazhou Bay Innovation Institute, Hainan Tropical Ocean University, Marine Food Engineering Technology Research Center of Hainan Province, Collaborative Innovation Center of Marine Food Deep Processing, Hainan Key Laboratory of Herpetological Research, Sanya 572000, China*

[b] *United Graduate School of Agricultural Sciences, Ueda 3-8-18, Morioka, Iwate 020-8550, Japan c Faculty of Agriculture, Iwate University, Ueda 3-8-18, Morioka, Iwate 020-8550, Japan*

[c] *School of Food Science and Engineering, Ningbo University, Ningbo 315800, Zhejiang, China*

[d] *College of Ocean Food and Biological Engineering, Jimei University, Xiamen 361021, China*

Corresponding author: Dongxue Wang

Tel.: +86-13578636252

Email: wangdongxue@hntou.edu.cn

Table S4. Experimental factors for response surface optimization

Level	Factors		
	A Rice starch（%）	B Deep-sea salt（%）	C Lutein（%）
-1	1.5	1.0	0.2
0	2.0	1.5	0.5
1	2.5	2.0	0.8

Table S5. Experimental design and results of response surface optimization

	A: Rice starch (%)	B: Deep-sea salt (%)	C: Lutein (%)	Y: Sensory score
1	-1	0	1	8.105
2	0	-1	-1	7.810
3	1	-1	0	8.060
4	0	-1	1	7.900
5	-1	0	-1	8.100
6	-1	1	0	8.040
7	1	0	-1	8.105
8	0	0	0	8.665
9	0	0	0	8.675
10	1	1	0	8.050
11	0	0	0	8.675
12	0	0	0	8.685
13	-1	-1	0	8.000
14	0	0	0	8.675
15	0	1	-1	7.890
16	1	0	1	8.115
17	0	1	1	7.875

Table S6. ANOVA results

Source	Sum of squares	df	Mean square	F-value	p-value	
Model	1.71	9	0.1896	1726.47	<0.0001	significant
A-starch	0.0007	1	0.0007	6.40	0.0392	
B-deep-sea salt	0.0009	1	0.0009	8.22	0.0241	
C-lutein	0.0008	1	0.0008	7.28	0.0307	
AB	0.0006	1	0.0006	5.69	0.0485	
AC	<0.0001	1	<0.0001	0.0569	0.8183	
BC	0.0023	1	0.023	20.54	0.0027	
A^2	0.1705	1	0.1705	1552.82	<0.0001	
B^2	0.8013	1	0.8013	7296.59	<0.0001	
C^2	0.5687	1	0.5687	5178.02	<0.0001	
Residual	0.0008	7	0.0001			
Lack of Fit	0.0006	3	0.0002	3.79	0.1153	not significant
Pure Error	0.0002	4	0.0001			
Cor Total	1.71	16				
Std. Dev	0.0105		R^2		0.9995	
Mean	8.20		Adjusted R^2		0.9990	
C.V.%	0.1278		Predicted R^2		0.9945	
Press			Adeq Precision		105.5215	
